# R. G. Poole Lansdown

**Published:** 1924-01

**Authors:** 


### Obituary.

R. G. Poole Lansdown, M.D., B.S., died suddenly on February 17th. As this issue had already gone to press, we are unfortunately restricted to a bare announcement of the deplorable loss of our much-beloved colleague until the forthcoming March number appears.

